# Game-Based Learning in Neuroscience: Key Terminology, Literature Survey, and How To Guide to Create a Serious Game

**DOI:** 10.1212/NE9.0000000000200103

**Published:** 2023-11-29

**Authors:** Sarah L. Edwards, Eric Gantwerker, Michael Cosimini, Alison L. Christy, Angel W. Kaur, Ann K. Helms, Mikaela L. Stiver, Zachary London

**Affiliations:** From the Emergency Department (S.L.E.), University Hospitals of Nottingham NHS Trust, United Kingdom; Department of Otolaryngology–Head and Neck Surgery (E.G.), Donald and Barbara Zucker School of Medicine at Hofstra/Northwell, New York, NY; Oregon Health and Science University (M.C.); Providence Health and Services (A.L.C.), Providence-St. Vincent Pediatric Specialty Clinic, Portland, OR; University of North Carolina (A.W.K.), Chapel Hill; Department of Neurology (A.K.H.), Medical College of Wisconsin, Milwaukee; Division of Anatomical Sciences (M.L.S.), Department of Anatomy and Cell Biology, McGill University, Montreal, Quebec, Canada; and @zach_london (Z.L.).

## Abstract

Game-based learning (GBL) has emerged as a promising approach to engage students and promote deep learning in a variety of educational settings. Neurology and neuroscience are complex fields that require an understanding of intricate neural structures and their functional roles. GBL can support the acquisition and application of such knowledge. In this article, we give an overview of the current state of GBL in neuroscience education. First, we review the language of gaming, establishing conceptual definitions for game elements, gamification, serious games, and GBL. Second, we discuss a literature review of games in the educational literature for adult learners involved in neuroscience. Third, we review available games intended for neuroscience education. Finally, we share tips for educators interested in developing their own educational games. By leveraging the unique features of games, including interactivity, feedback, and immersive experiences, educators and learners can engage with complex neuroscience concepts in a fun, engaging, and effective way.

## Introduction/Background and Terminology

The field of neuroscience (defined here to encompass all aspects of neuroscience, neurology, and neuroanatomy) presents unique challenges for educators and learners. The concepts of localization and the complexity of neural structures and related functional mechanisms can be daunting. Medical students perceive neurology as the most difficult discipline in medicine and report more anxiety when it comes to generating neurology-related differential diagnoses compared with other specialties.^1,2^

Educational games offer a promising solution to some of these challenges. Games and game elements can be used in education in a variety of ways: to deliver content, introduce concepts, solidify learned information, and/or modify learner behaviors.^3,4^ Educational gameplay can promote engagement, improve learning outcomes, facilitate the development of critical thinking skills, and encourage collaboration and teamwork skills when compared with traditional didactics.^5^ One contributor to these outcomes could be increased self-determined motivation experienced by learners as they engage in the game activity. This concept draws from the self-determination theory of motivation, which posits that learners experience greater intrinsic motivation when their basic psychological needs for autonomy (to direct one's own behavior), competence (to feel effective in one's actions), and relatedness (to feel connected with others) are met.^6^

The literature on neuroscience game-based learning (GBL) is limited; however, games outside of the neuroscience field have been shown to have positive educational outcomes such as increased application of physiology concepts, and potentially diagnostic ability, in a clinical setting and improved test performance.^7,8^ These findings suggest that the use of games in neuroscience education could provide students with a fun and engaging activity that can improve content understanding and learning, making this a promising area of future research. Using games in neuroscience education could help students see themselves in a career in neurology or neuroscience, potentially contributing to remedying projected workforce deficits in clinical neurology.^9,10^ This study aims to give neuroscience educators an introduction and overview of the utility of GBL within the field.

## Definitions

As with any rapidly changing area of research, working definitions of terms within the field of educational games are subject to change and open to interpretation. Below we define the key terms because we will use them through this article: game elements and core game loop, gamification, serious games, and GBL. We encourage other researchers in this space to provide working definitions at the beginning of their articles to contextualize the work in the greater landscape of educational games.

### Game Elements and the Core Gameplay Loop

Game elements (or game mechanics) are the component parts or working pieces of games that facilitate the interactions, motivations, and entertainment that accompany gameplay.^11^ Games are built on what is termed the *core gameplay loop*, in which players follow rules to interact with the game, receive feedback, and either progress in the game or repeat the loop with slight modifications to their actions.^11^ For example, in the game Pac-Man, the player directs Pac-Man to eat dots and score points until either the level is complete or Pac-Man hits a ghost and dies. Games take this core gameplay loop and build on it by adding other game elements and mechanics such as competition through leaderboards (a visual representation of how participants rank in descending order), badges (virtual rewards or symbols that users earn when they achieve a certain milestone or complete a specific task), and social interaction (ways in which players can communicate or engage with each other).^11^

### Gamification

The term “gamification” typically refers to the integration of game elements into nongame settings.^3^ These game elements (e.g., competition, scoring, and attaining badges) can be applied to a broad range of contexts, including tracking steps, buying coffee, or interacting with airlines. In the field of education, gamification has been purported to modify learner behaviors and enhance motivation. For example, the application of gamification in the form of a team competition for points led to increased attendance at surgical conferences.^12^ In neurology, the *Question of the Day* mobile app developed by the American Academy of Neurology uses game elements such as competition, leaderboards, and daily streaks within a board-style multiple-choice question format to encourage consistent participation in the educational activity.^13^

Gamification may lead to increased satisfaction, engagement, and motivation in medical learning^14,15^ and specifically in neuroscience education; however, some studies have not found psychological benefits with gamification,^16^ possibly due to variability of game elements and/or participants.

### Serious Games

*Serious games* are designed without entertainment as the main purpose or intent. The goal of these games is usually educational, with learning often being prioritized over fun. Serious games encourage active experiential learning and have made inroads in many industries, including undergraduate and health profession education. When playing serious games, players must engage with the core gameplay loop to receive feedback and understand the consequences of different actions. The resulting cycle of judgment, behavior, and feedback drives home learning objectives, provides educational content, and reinforces motivation to participate. We view the game cycle as iterative, such that gameplay involves repeated judgment-behavior-feedback loops. That is, gameplay can lead to certain user judgments or reactions such as increased interest, enjoyment, involvement, or confidence; these reactions lead to behaviors such as greater persistence or intensity of effort; and these behaviors result in system feedback on performance in the game context.^11,17^

Current research suggests that games are most effective at promoting learning gains when educational content is integrated into the core mechanics of the game.^17^ Serious games have performed better than traditional didactics for assessments in randomized studies for pharmacology^8^ and physiology.^7^ In neurology specifically, *Stroke of Genius* is a neuroscience-themed serious card game in which players formulate an evolving and convincing story for a stroke syndrome that relates to cards in play.^18^ Each card represents a facet of neuroanatomy, a stroke risk factor, or a pathophysiologic process.^19^ These mechanics require students to engage in both divergent and convergent thinking within the context of content knowledge with support from the facilitating instructor.

### Game-Based Learning

GBL is another term with varying definitions. Some authors use it to mean only educational gaming in a digital space,^20^ while others use it more broadly to encompass all learning involving games, including gamification.^21^ It may be easiest to differentiate the use of GBL, serious games, and gamification in education by considering how game elements are used and the primary goal of the game (Table 1). Our preference is to use the term GBL broadly to encompass all games that aim to educate (serious or otherwise) regardless of format, following the approach of Plass et al.^22^

**Table 1:** Comparison of Games, Gamification, Serious Games, and Game-Based Learning

	Game	Gamification	Serious games	Game-based learning
Examples	*Super Mario Brothers*, *Catan*, *Cranium*, *Chess*	*Neurology Question of the Day App*	*NeuroNavigator*, *The Plexus*, *Cerebro*, *Stroke of Genius*	*Foramina!*, *Endowed Chairs*
Definition	A system where players engage in an artificial conflict defined by rules that results in a quantifiable outcome^51^	Application of game elements to a nongame context (not exclusive to education)^3^	A game designed for a purpose other than entertainment (not exclusive to education)^54^	The use of games in service of educational purposes (inclusive of serious games)^4^
Goal	Creating meaningful play^51^	Modify learners' behaviors or attitudes in service of an educational goal	Modify learners' knowledge, skills, or attitudes by using the core game loop of a game designed for that purpose	Facilitate learning through the use of games
Format	Wide variety of formats including analog and digital experiences	Often digital in the form of web-based or application-based point systems, leaderboards, or badges	Often stand-alone digital or tabletop games	Includes use of serious games, commercial games, role-playing games, or other games specific to the classroom setting
When to use	Commonly used for entertainment purposes, both individually and in a social setting. Can be used for team building or wellness in an educational setting	Suitable for situations where the objective is to enhance engagement with existing teaching materials	Useful when a creative game solution can effectively align with the learning objectives or demonstrate specific content, as well as practice particular skills	A methodology that integrates game mechanics with learning objectives in a customized solution

Unlike serious games, GBL can still occur in games where the primary focus is entertainment. In *Monopoly*, for instance, players roll dice and buy properties in hopes of driving their opponents to bankruptcy. The goal is entertainment (not education), but with experience, players may start to remember the names of the properties, their color grouping, what order they were in, and may even recall how much they cost. In this way, learning is facilitated as a byproduct of fun; however, the game can also be used to intentionally promote learning, In fact, *Monopoly* has been used to teach diverse topics from accounting to sociology based on the mechanics and theme of the game.^23^ The details of the game can be altered to repurpose it for an educational context, referred to as “purpose shifting.”^24^ If the details of the *Monopoly* properties were modified to incorporate health profession education–related content, players could acquire relevant knowledge through gameplay. This altered game would enable medical GBL.

*Foramina!* is an example of a neuroscience game that can be used for GBL, despite a primary focus on entertainment.^25^ It is a cranial nerve–themed tabletop game in which players roll dice, collect axons (as a form of currency), and buy cards. The strategy depends more on understanding the probabilities of different dice rolls than on a prior knowledge of neuroanatomy. However, players may become familiar with the names, numbers, functions, and some anatomical features of the cranial nerves as a side benefit of play.

## Current GBL Literature

Our team performed a systematic search of literature on March 23, 2023, using the following databases: MEDLINE, Embase, and CINAHL. We collaboratively established most of the inclusion and exclusion criteria for this systematic search prior to the first stage of article screening with minor adjustments made throughout. The final inclusion criteria were as follows: (1) any primary literature involving a serious physical game, including innovation reports and conference proceedings; (2) nondigital (i.e., tabletop) format; (3) audience was any learner undergraduate, graduate, and postgraduate education related to neurology, neuroscience, or neuroanatomy; and (4) published in any language. We excluded articles for which any of the following criteria applied: (1) games with a fully digital or online format; (2) interventions that only role-play or simulation in a nongame context (e.g., simulated patients, role-play scenarios); and (3) games tested exclusively with students who were not undergraduate, graduate, or postgraduate (e.g., high school students).

A total of 2,068 articles were initially screened for inclusion (S.L.E. and M.L.S.). After removing the duplicates and reviewing the titles and abstracts according to the aforementioned eligibility criteria, we were left with 47 full texts. After independent review of the full texts (S.L.E. and M.L.S.) and discussion with the rest of the authors, we found 13 studies that were eligible for inclusion. Of the 13 studies, 7 were original research^26-32^ and 6 were conference abstracts.^19,33-37^ Studies were published as early as 2015^30-32^ and as recently as 2023.^29^ The summary of the papers included are listed in eTable 1 (links.lww.com/NE9/A52). Six studies described card games,^19,28,29,35,38,39^ 6 described board games,^26,27,30-32,37^ and 1 described a puzzle game.^36^ Most of the games were created in English (n = 6)^19,26,30-32,35^ or Portuguese (n = 4).^35,36,38,39^ The identified games covered a variety of themes, with basic science, clinical neurology, and neuroanatomy being the most popular. A common objective among the games described in the literature was to enhance knowledge retention by providing a unique and engaging approach to reviewing previously learned content. For instance, Kaur^26^ highlighted the effectiveness of a specific game in achieving this goal. Educational outcomes reported in the articles primarily focused on improvement of knowledge as measured by preintervention and postintervention assessments.^26,28,29^

Educational gaming is fun, thought-provoking, and engaging, but it can also provide benefits beyond increasing retention of content knowledge. Decreasing stress, fatigue, and burnout can improve medical education and increase physician well-being.^40^ We did not find any studies in our literature search specifically addressing whether games improve wellness in medical school, residency, or in any aspect of neuroscience education. However, we know games are generally accepted to be fun and enjoyable, and promote community building among participants. Future research could aid our understanding of the utility of games to promote wellness in neuroscience education.

## Neuroscience Games Currently Available for Use

Using existing games can decrease the time required for preparing engaging and motivating educational materials, alleviating the workload of neuroscience educators. To support educators seeking to incorporate GBL into their courses, we identified neuroscience games currently available for use.

Among the games described in the literature, 3 tabletop games are currently available for purchase or play by others,^26,28,29^ and several identified digital games are no longer usable.^41-43^ To complement our search of published literature, we also searched for games available for learners in undergraduate, graduate, or medical education. In November 2022, we searched crowdfunding services, print-on-demand services, a board game database, and catalogs of publishers of card and board games. Search terms included “neurology,” “neuroscience,” “neuroanatomy,” “education,” and “educational.” In early 2023, we searched additional websites dedicated to games (e.g., BoardGameGeek.com), sites specific to educational and medical education games (e.g., Focus games, Nerdcore Medical, Genius games), MedEdPortal, and digital gaming platforms (Steam, App Store, Google Play). In total, we identified 83 games with potential for inclusion and further refined this list to exclude games that were no longer available or usable, unsuccessfully crowdfunded, or lacked true game elements. We then contacted the game designers to confirm details of these games and requested recommendations for other games. At the end of this process, 14 available games were included in this review, and a full list of these games is in the supplementary eAppendix 1 (links.lww.com/NE9/A51).

Of these 14 games, the most common method of game publication was print-on-demand (n = 10). Most of the currently available games (n = 11) were published in 2020 or later. Medical students were the most frequent intended audience (n = 8), and neuroanatomy was the most common topic (n = 6). The emphasis is well-justified, given the complexity of neuroanatomy for many students and the suitability of game-based adaptations due to the visual representation of neuroanatomic structures during gameplay.

The reviewed games use a variety of mechanics to match their respective educational goals and often repurposing well-known party game mechanics. For example, *Forbidden Neurds*^44^ uses *Taboo*-like mechanics, where players must communicate a neuroscience term without using specific “forbidden” words. The *Neurological Hat Game* uses mechanics akin to *Celebrity*, requiring players to describe a symptom with full phrases, then a single word, and finally with mime.^28^ Other games use navigation mechanics; *NeuroNavigator*^45^ divides neuroanatomy into a grid to help players learn the relative positions of structures, while *Cerebro*^46^ uses a navigation mechanic in a digital format. In *The Plexus*,^47^ players score points for collecting cards mapping the pathways of neurons traveling through the brachial plexus.

Beyond the games reviewed earlier, many residency programs have created their own trivia games, including “QuizBowl” or “Neuro Jeopardy,” to increase engagement and socialization in a learning environment.^48^ These games, whose mechanics are often based on *Jeopardy* or *Trivial Pursuit*, offer an engaging framework for retrieval practice. Games designed with more complex mechanisms to require transfer, application, or manipulation of information can further boost their educational value.

## Making Your Own Educational Game

Entering into game development can be daunting, but rewarding. The process can be thought of in a process somewhat analogous to Kern's 6 steps for curriculum development,^49^ though generally we would encourage early experimentation to avoid excessive work prior to testing of a game design. Based on our own experience with game development and review of processes of games developed for education of health professionals,^18,50-52^ we would suggest starting with the following questions:

1. *What are the educational goals?* Start by establishing clear and specific educational goals and learning outcomes. Focus on something easily definable, such as a specific area of neuroanatomy, a diagnosis, or a single group of diseases and their treatments. Decide whether the game will teach new material in place of a lecture or reinforce previously learned knowledge.
2. *Who is the intended audience?* Is your game for undergraduates, preclinical medical students, neurology clerkship students, residents, or practicing clinicians? Use available educational resources to help target your content to the appropriate level of learners. Be intentional about your goals, whether you plan to engage your own learners or distribute your game to a broader audience. This can also help you identify what prior knowledge (if any) is required to play the game.
3. *What mechanics will work best?* Start by playing as many games as you can and pay attention to the mechanics that you enjoy. How could these mechanics translate to your learning goals? You can take mechanics directly from existing games; you can copyright a game, but not the game mechanics! For instance, *Cards Against Humanity* uses the same basic mechanics as *Apples to Apples*, and the neuroanatomy-themed game *Foramina!* has a similar core gameplay loop to *Machi Koro*. For more inspiration, consider seeking out videos, podcasts, books, or seminars about game development.
4. *What format do you want to use?* Tabletop games (board, card, or dice game) are intrinsically social, relatively inexpensive to create, and do not become obsolete due to changes in technology. However, the educational content of tabletop games, except for downloadable “print-and-play” tabletop games, is more difficult to update than that of digital games. Digital games tend to be daunting, but today's software advances allow for more user-friendly ways to dip your toe into this space.
5. *How will your learners interact with the game?* Think about how many learners can play at once and whether a content expert is needed to facilitate throughout the game. Determine how complex the game will be, keeping in mind that the time needed to explain rules may cut into the time available to play. In addition, remember that learners have different levels of experience and comfort with tabletop games. More rules and greater strategic complexity may engage some learners while alienating others. Designing games to meet your identified learners’ autonomy, competence, and relatedness needs would help create games that more effectively engage and motivate them through gameplay. It also may be helpful to think how long the game is intended to take whether it be a few minutes or up to an hour or more.
6. *How do I refine my game to make sure it works?* Games are most effectively refined through playtesting, which can be performed using a simple prototype created with household office supplies. If your game is more complicated, break it into steps or sections to test each part and then adjust or modify them separately to better fit your game's overarching goal until they are ready to be tested as a whole. Include both experienced gamers and members of your target audience in playtesting at various stages of game development. In addition to soliciting feedback, take time to observe gameplay to identify what moments spark excitement, frustration, or confusion.
7. *How professional should my final game production be?* Consider the look and feel of the game. If you are creating a serious game and plan to use it as part of a curriculum, a low-tech option may be ideal. If you are hoping to appeal to a wider audience, you may consider creating or commissioning high-quality graphics and using more expensive game components. In theory, visually arresting graphic design elements could facilitate learning through multimodal stimulation, but the degree to which it bolsters the educational value of the activity is unknown. Ultimately, you will have to consider whether commissioned graphic design and premium game components add enough to your game to justify the additional design and production costs.
8. *How do I measure the success of a game?* Measures of success will be drawn from the goals you set for your game in step 1. A large proportion of educational materials measure success through educator and learner surveys assessing enjoyability and perceived efficacy at materials and skills acquisition. A more rigorous assessment can be achieved through before and after comparison of learner performance, such as pretesting and posttesting of learners on the material presented, and comparing performance between a group playing the game and learners engaged in more traditional educational activities. One could use already extant tests, such as the Residency In-service Training Examination,^53^ or specifically design a test for the comparison groups to take.

## Conclusion

The field of neuroscience offers a captivating and intricate landscape for game development, presenting an exciting opportunity to engage in this domain. Over the past decade, there has been a surge in research articles focused on GBL in neuroscience. As we delve deeper into this field, there is a growing need for a wider array of games, both tabletop and digital, and additional studies to explore how different game mechanics can enhance comprehension across various aspects of neuroscience. Future work could include seeing whether there is any impact of benefit of GBL in the neuroscience field, encompassing areas such as anatomy, clinical practice, population-based care, neuroethics, and provider wellness. We eagerly await the advancements and discoveries that the forthcoming decade will bring to this dynamic realm.

## Appendix Authors

**Table:**

Name	Location	Contribution
Sarah L. Edwards, BMBS, MSc (PEM), MSc (Med Ed), FHEA	Emergency Department, University Hospitals of Nottingham NHS Trust, United Kingdom	Drafting/revision of the article for content, including medical writing for content; major role in the acquisition of data; study concept or design; and analysis or interpretation of data
Eric Gantwerker, MD, MMSc (MedEd)	Department of Otolaryngology–Head and Neck Surgery, Donald and Barbara Zucker School of Medicine at Hofstra/Northwell, New York, NY	Drafting/revision of the article for content, including medical writing for content; major role in the acquisition of data; study concept or design; and analysis or interpretation of data
Michael Cosimini, MD	Oregon Health and Science University, Portland	Drafting/revision of the article for content, including medical writing for content; major role in the acquisition of data; study concept or design; and analysis or interpretation of data
Alison L. Christy, MD, PhD	Providence Health and Services, Providence-St. Vincent Pediatric Specialty Clinic, Portland, OR	Drafting/revision of the article for content, including medical writing for content; major role in the acquisition of data; study concept or design; and analysis or interpretation of data
Angel W. Kaur, PhD	University of North Carolina, Chapel Hill	Drafting/revision of the article for content, including medical writing for content; major role in the acquisition of data; study concept or design; and analysis or interpretation of data
Ann K. Helms, MD, MS	Department of Neurology, Medical College of Wisconsin, Milwaukee	Drafting/revision of the article for content, including medical writing for content; major role in the acquisition of data; study concept or design; and analysis or interpretation of data
Mikaela L. Stiver, PhD, MSc	Division of Anatomical Sciences, Department of Anatomy and Cell Biology, McGill University, Montreal, Quebec, Canada	Drafting/revision of the article for content, including medical writing for content; major role in the acquisition of data; study concept or design; and analysis or interpretation of data
Zachary London, MD	Department of Neurology, Division of Neuromuscular Medicine, University of Michigan School of Medicine, Ann Arbor	Drafting/revision of the article for content, including medical writing for content; major role in the acquisition of data; study concept or design; and analysis or interpretation of data

## Glossary

GBL: game-based learning
